# Epidemiological, Clinical, and Phylogenetic Characteristics of the First SARS-CoV-2 Transmission in a Nursing Home of Singapore: A Prospective Observational Investigation

**DOI:** 10.3389/fmed.2021.790177

**Published:** 2022-01-28

**Authors:** Junxiong Pang, Huei Nuo Tan, Tze Minn Mak, Sophie Octavia, Sebastian Maurer-Stroh, Fernanda L. Sirota, Mark Peng Chew Chan, Ian Yi Onn Leong, Valerie T. J. Koh, Peng Lim Ooi, Shawn Vasoo, Dale Fisher, Lin Cui, Heidi Rafman, Jeffery Cutter, Vernon J. Lee

**Affiliations:** ^1^Ministry of Health, Singapore, Singapore; ^2^Saw Swee Hock School of Public Health, National University of Singapore and National University Health System, Singapore, Singapore; ^3^Department of Geriatric Medicine, Tan Tock Seng Hospital, Singapore, Singapore; ^4^National Public Health Laboratory, National Centre for Infectious Diseases, Singapore, Singapore; ^5^Bioinformatics Institute, Agency for Science, Technology and Research, Singapore, Singapore; ^6^Department of Biological Sciences, National University of Singapore, Singapore, Singapore; ^7^Genome Institute of Singapore and Bioinformatics Institute, Agency for Science, Technology and Research, Singapore, Singapore; ^8^Division of Central Health, Tan Tock Seng Hospital, Singapore, Singapore; ^9^National Public Health and Epidemiology Unit, National Centre for Infectious Diseases, Singapore, Singapore; ^10^Yong Loo Lin School of Medicine, National University Hospital and National University Health System, Singapore, Singapore; ^11^Agency for Integrated Care, Singapore, Singapore

**Keywords:** public health, epidemiology, genomic epidemiology, nursing home, infection prevention and control

## Abstract

Severe acute respiratory syndrome coronavirus 2 (SARS-CoV-2) transmission has resulted in a significant burden among nursing home facilities globally. This prospective observational cohort study aims to define the potential sources of introduction and characteristics of SARS-CoV-2 transmission of the first nursing home facility in Singapore. An epidemiological serial point-prevalence survey of SARS-CoV-2 was conducted among 108 residents and 56 healthcare staff (HCS). In the current study, 14 (13%) residents and two (3.6%) HCS were diagnosed with coronavirus disease 2019 (COVID-19), with a case fatality rate (CFR) of 28.6% (4/14) among the residents. The median age of the infected residents was 86.5 [interquartile range (IQR) 78.5–88] and 85.7% were women. Five residents were symptomatic (35.7%) and the others were asymptomatic (64.3%). A higher proportion of residents who succumbed to COVID-19 had hypertension than those who recovered. The SARS-CoV-2 whole-genome sequencing showed lineage B.6 which is rare globally but common regionally during the early phase of the pandemic. Household transmission is a potential source of introduction into the nursing home, with at least six epidemiologically linked secondary cases. Male residents were less implicated due to the staff segregation plan by block. Among residents, a higher proportion of the non-survivors were asymptomatic and had hypertension compared with survivors.

## Significance of This study

What is already known about this subject?

- Nursing homes have been associated with high mortality and morbidity due to COVID-19 disease.
- A high level of infection prevention and control is important to reduce the risk of transmission.
- The source of introduction is usually challenging to determine.

What are the new findings?

- Infected residents were largely asymptomatic.
- Residents who succumbed to COVID-19 were largely asymptomatic and had hypertension.
- Healthcare staff has a lower risk of infection as compared with residents.
- Phylogenetic analyses of the viral genetic material from infected staff suggested staff who stayed outside the nursing home facility as a potential source of introduction.

How might these results change the focus of research or clinical practice?

- Active surveillance for SARS-CoV-2 infection among residents and nursing staff in a nursing home is critical to reducing the risk of an introduction and transmission.
- Reducing the number of residents per ward and per HCS would reduce the risk of transmission.

## Introduction

Enhanced surveillance was activated across the whole public health system in Singapore since the reported cluster in Wuhan, China in December 2019. The first imported and locally transmitted coronavirus disease 2019 (COVID-19) cases detected in Singapore were on January 23 and February 1, 2020, respectively. Although a slew of public health measures, such as active contact tracing, quarantine of close contacts, and travel restrictions were implemented, the number of community cases started to increase significantly toward the end of March. To reduce transmission of the virus in the community, Singapore introduced a modified lockdown known as “circuit breaker,” which was enforced from April 7 to June 1, 2020. Post-lockdown on June 9, 2020, there were about 39,000 cases and 25 deaths reported in Singapore.

Nursing homes have been associated with high mortality and morbidity in many countries due to the novel severe acute respiratory syndrome coronavirus 2 (SARS-CoV-2) virus, which resulted in the COVID-19 (1–4). Nursing homes are communal living facilities with a vulnerable population that requires rendered care, tends to be older, and has multiple comorbidities, cognitive and physical disabilities. They are a hotbed for large outbreaks because of difficulties in the detection and implementation of safe distancing and infection control measures (5, 6).

Nursing homes in Singapore are long-term care facilities catering to those who require skilled nursing care, medical care, and rehabilitative services. There are 77 nursing homes (40% privately run, remaining publicly owned or not for profit) with a total of 16,221 beds (24% of beds are in private nursing homes, remaining in publicly owned or not-for-profit nursing homes). The average nursing home size is about 250 beds but ranges from 16 to 624 beds (in 2020). Very few residents have private rooms; almost all residents stay in shared rooms with 4 or more beds. There are always nursing and care staff on hand, with regular consultations provided by general practitioners, specialists, and allied health professionals. Although the incidences of COVID-19 from nursing homes are very low during the early phase of the pandemic in Singapore (7, 8), it is still important to learn from every nursing home cluster that had occurred. In this study, we aim to identify the potential source of infection and characteristics of SARS-CoV-2 transmission through a combination of clinical, epidemiological, laboratory, and phylogenetic approaches.

## Methods

### Serial Point-Prevalence Survey (PPS)

A serial point-prevalence survey (PPS) among the residents was performed to assess the extent of transmission. For the initial PPS, nasopharyngeal swabs were performed on all residents of the facility and healthcare workers (HCWs) who were symptomatic. During any other time, residents and HCWs who were symptomatic were conveyed to an acute hospital for further evaluation to rule out COVID-19. Individuals who had positive swab PCR results were immediately admitted to the hospital for isolation and further management.

### Coronavirus Disease 2019 Laboratory Diagnosis, Sequencing, and Phylogenetic Investigation

All primary samples tested PCR positive for SARS-CoV-2 by reverse transcription PCR (RT-PCR) at diagnostic laboratories were forwarded to the National Public Health Laboratory, under the provisions of the Infectious Diseases Act in Singapore for testing and validation using an in-house method as previously described (9). Briefly, three specific real-time RT-PCR methods targeting the N, S, and ORF1ab genes were designed to detect the presence of SARS-CoV-2 in clinical samples. The primer sequences for the N gene are: forward primer 5′ CTC AGT CCA AGA TGG TAT TTC T; and reverse primer 5′ AGC ACC ATA GGG AAG TCC. The probe sequence is 5′ FAM-ACC TAG GAA CTG GCC CAG AAG CT-BHQ1. The sequences for the ORFlab real-time RT-PCR are: forward primer 5′ TCA TTG TTA AYG CCT ATA TTA ACC; reverse primer: 5′ CAC TTA ATG TAA GGC TTT GTT AAG; and probe: 5′ FAM-AAC TGC AGA GTC ACA TGT TGA CA-BHQ1. The sequences for the S gene real-time RT-PCR are: forward primer 5′ TAT ACA TGT CTC TGG GAC CA; reverse primer 5′ ATC CAG CCT CTT ATT ATG TTA GAC; Probe: 5′ FAM-CTA AGA GGT TTG ATA ACC CTG TCC TAC C-BHQ1. Thermal cycling for N gene real-time RT-PCR assays was performed at 50°C for 20 min for reverse transcription, 95°C for 15 min, 50 cycles of 94°C for 5 s, 55°C for 1 min. Thermal cycling for both ORF1ab and S gene real-time RT-PCR assays were performed at 50°C for 20 min for reverse transcription, 95°C for 15 min, 50 cycles of 94°C for 5 s, 50°C for 20 s, and 72°C for 20 s.

Residual RNA was subjected to tiled amplicon PCR using ARTIC nCoV-2019 version 3 panel (10), where One-Step RT-PCR was performed using the SuperScript™ III One-Step RT-PCR System with Platinum™ *Taq* DNA Polymerase (Thermo Fisher Scientific, MA, USA). Sequencing libraries were prepared using the Nextera XT and sequenced on MiSeq (Illumina, CA, USA) to generate 300 bp paired-end reads. The reads were subjected to a hard-trim of 50 bp on each side to remove primer artifacts using BBMap prior to consensus sequence generation by Burrows-Wheeler Aligner-MEM v0.7.17, with default settings (11). The generated consensus sequences were shared *via* a global initiative on sharing avian flu data (GISAID) (12). Closely related representative strains from other countries (99.99% identity and matching the time window) were identified in the GISAID database using BLASTN (13). Due to the epidemiological context of travel from Sri Lanka for one case, the three phylogenetically closest sequences in GISAID from Sri Lanka were included. All sequences were aligned using MAFFT (v7.427) (14), with hCoV-19/Wuhan/WIV04/2019 (accession: EPI_ISL_402124) as a reference together with CoV-19/Singapore/1/2020 (EPI_ISL_406973) to root the tree. The alignment was manually inspected and trimmed using Jalview (15). IQ-TREE v1.6.1 (16) was used with ModelFinder (17) and 1,000 step ultrafast bootstrapping (18) to create a maximum likelihood phylogenetic tree with zero length branches collapsed. A total of 41 sequences were reannotated with the latest Pango versions (updated regularly on GISAID) and it shows the majority fall into lineage B.6.6 (which became more detailed from B.6 previously after the Pango update) and the GISAID clades remain the same. The CoVsurver was used to calculate all amino acid mutations and if they occur in relevant sites with known phenotypes. None of them appear relevant from a possible functional perspective (Supplementary Material).

### Statistical Analysis

Descriptive analysis was conducted using median and interquartile range (IQR) for continuous data, the percentage for categorical data. Chi-square and *t*-tests were performed for categorical and continuous data, respectively. The value of *p* = 0.05 was used as the cut-off for statistical significance using STATA software (Stata Corp LLC, TX, USA).

### Ethics Declaration

This study was conducted in response to a national public health emergency. All above-mentioned activities and measures were performed in accordance with the guidelines and guidance of the laboratory safety, infection prevention and control, and clinical management circulars approved by the Ministry of Health (MoH) for the management of the COVID-19 outbreak as well as the Infectious Diseases Act enacted by Parliament in Singapore to safeguard the public health in Singapore. Informed consent was obtained from all subjects under the Infectious Diseases Act.

## Results

### Epidemiological Investigation and Responses

**Figure 1:**
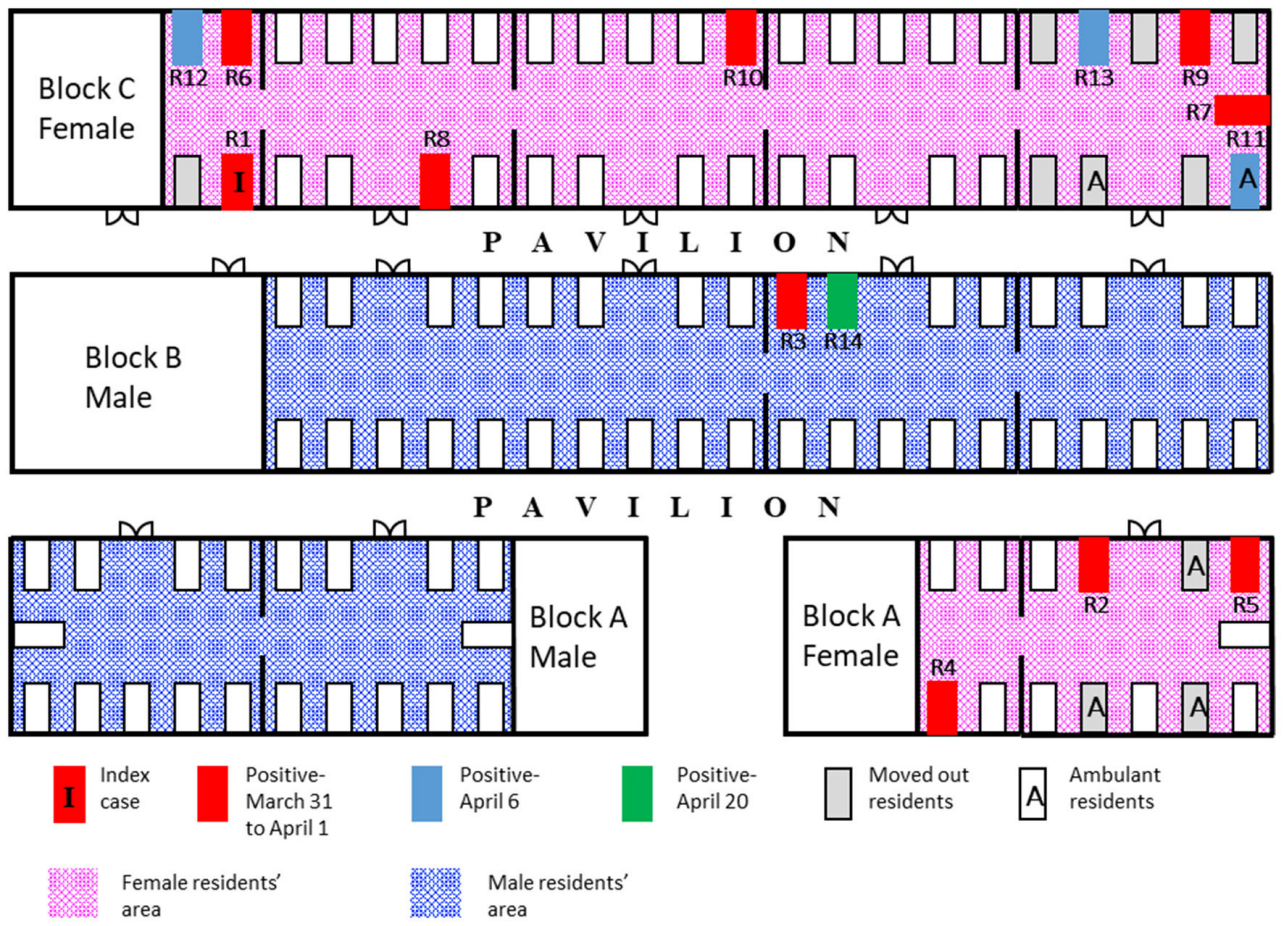
Spot map of the facility. Schematic diagram of the facility (not drawn to scale) showing the spot map of infected cases and date of diagnosis; residents who were moved out of the facility on day 8 (April 7) of the outbreak.

The nursing facility had a 111-bed capacity with 55 female residents, 53 male residents, and 56 HCWs at the time of the outbreak. The facility is housed across 3 single-level blocks—block A housed both men and women in two separate wings, block B housed only male residents, and block C had only female residents (Figure 1). Each block consists of 3–5 cubicles with 4–18 beds in each cubicle and the residential areas had natural ventilation with fans. Approximately 20% of the residents were ambulant. The remainder were non-ambulant or required assistance. All residents were of Chinese ethnicity.

On March 30, 2020, a female resident had developed a fever. The woman was conveyed to an acute hospital after the condition deteriorated despite having received treatment for presumed aspiration pneumonia. The woman was diagnosed with COVID-19 on March 31. Active surveillance at the facility and enhanced infection prevention and control practices (IPC) were implemented. The activities and contact tracing of each case in the last 14 days were performed to obtain information on the nature and duration of all possible exposure to the index case during the infectious period among the staff, visitors, and female residents using a standardized institutional contact tracing template. Possible epidemiological links between cases and their household members were derived and analyzed from interviews with nursing home staff and affected household members. An imported case was defined as a laboratory-confirmed case identified in Singapore with travel history from COVID-19 affected countries in the last 14 days before the onset of symptoms. The swabs for residents were performed on-site by a mobile team for PCR testing for SARS-CoV-2, which were completed by April 1, 2020. Thereafter, weekly point-prevalence surveys for asymptomatic residents were performed on April 6, 13, 20, and 24, 2020. All epidemiological investigations were implemented under the Infectious Diseases Act, and this included the use of data for analysis to guide public health interventions to control outbreaks.

The accommodation arrangements for the HCWs were reviewed to assess the risk of staff cross-infection. In accordance with the national policy enacted in February 2020, HCWs in the facility were segregated into different care teams based on the different blocks. The remaining residents of some of the affected wards were isolated in a vacant nursing home temporarily, after the second round of tests to minimize the ongoing transmission. Other residents chosen to be isolated were ambulant individuals with cognitive impairment who could not comply with movement restrictions (Figure 1). New admission or transfer of returning residents was suspended, except for returning residents who recovered from COVID-19 infection and were non-infectious.

With the identification of the index case, personal protective equipment (PPE) was stepped-up in patient care areas to add gloves and gowns, in addition to the pre-existing requirement for surgical masks. The N95 masks and eye protection were used when performing aerosol-generating procedures (such as suctioning and nasopharyngeal swabs) and when caring for residents with symptoms of acute respiratory infection. Alcohol-based hand sanitizers were strategically placed, and PPE donning and doffing locations were identified. The frequency of environmental cleaning of high-touch areas was increased. Nursing practice was changed to minimize the risk of cross-contamination from commonly used items, equipment, and supplies.

### Demographic and Clinical Characteristics of SARS-CoV-2 Infected Cases

**Figure 2:**
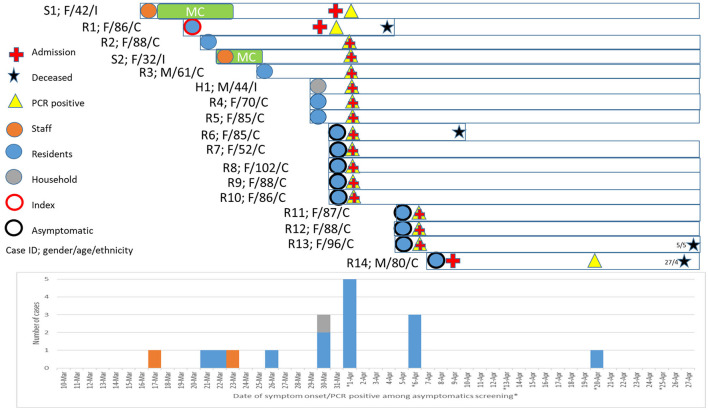
Epicurve and characteristics of coronavirus disease 2019 (COVID-19) cases. Epicurve was charted based on the onset of symptoms and swab positive notification from the serial point-prevalence survey. Gender, age, ethnicity (C, Chinese; M, Malay; and I, Indian), symptomatic status, date of PCR test, admission, and fatality were highlighted. The index and primary cases of the nursing home outbreak are R1 and S1, respectively. H1 is the household member of S1.

Among the 108 residents and 56 HCWs in the nursing home, 14 (13%) residents and two (3.6%) HCWs were diagnosed with COVID-19, corresponding to an overall attack rate of 9.8% (16/164). A higher proportion of residents who succumbed to COVID-19 had hypertension than those who recovered. The median age of the infected residents was 86.5 years (IQR 78.5–88) and 85.7% were women. Table 1 shows the characteristics of the infected residents and HCW. Nine residents and two HCWs (S1 and S2), excluding the index case, were diagnosed within the first 2 days of the outbreak. The two HCWs (S1 and S2) were women, of Indian nationality, in their early 40s and 30s years of age, respectively, and were taking care of the female wards. Three other residents were diagnosed on April 6 and 1 resident, R14, was diagnosed on April 20 (Figure 2). The last diagnosed resident was transferred to an acute hospital on Day 10 (April 9) of the outbreak for non-respiratory symptoms and was isolated throughout the whole hospitalization. The resident was asymptomatic prior to being diagnosed on April 20. Other than the index case, four other residents were symptomatic (35.7%) and the rest were asymptomatic (64.3%) before the time of the tests (Figure 2). However, four residents were found to be febrile on the day of admission (range 37.6–38.2°C) and 2 others developed a fever after the admission. Two infected residents had poor oral intake and hypoactive delirium with no respiratory symptoms. Eight of the infected residents were non-ambulant.

**Table 1:** Characteristics of nursing home residents and healthcare workers (HCWs) with coronavirus disease 2019 (COVID-19).

	**Residents**			**HCW** **(*n* = 2)**
	**Survivor** **(*n* = 10)**	**Non-survivor** **(*n* = 4)**	**All** **(*n* = 14)**	
**Demographics**				
Age, median (IQR) years	86.5 (67.8–88)	85.5 (81.3–93.5)	86 (77.5–88)	37
Female, No. (%)	9 (90)	3 (75)	12 (85.7)	2 (100)
**Comorbidities and function**				
Hypertension∧	3 (30)	3 (75)	6 (42.9)	0
Diabetes mellitus	0	0	0	0
Coronary heart disease	0	0	0	0
Cerebrovascular disease	2 (20)	0	2 (14.3)	0
Chronic respiratory disease	0	0	0	0
Chronic renal disease	2 (20)	1 (33.3)	3 (21.4)	0
Cognitive impairment	6 (60)	3 (75)	9 (64.3)	0
Ambulant	1 (10)	0	1 (7.1)	2 (100)
**Outcome**				
Symptomatic	4 (40)	1 (25)	5 (35.7)	2 (100)
Duration of admission, median (range) days	21 (12–31)	14 (5–25)	19.5 (12.8–28.3)	(9, 5)

∧ *p-value < 0.05*.

The cubicle with the highest number of cases was in block C (Figure 1). This cubicle had residents who were ambulant and had shouting behavior. Four residents, including the index case, died from COVID-19, resulting in a case-fatality rate of 28.6%. They were 80, 85, 86, and 96 years old, and died between 9 and 25 days from the date of diagnosis. All the others only had a mild illness. The outbreak was declared closed on April 24, 14 days after the last affected case had been transferred out of the facility.

Among the staff, 18 lived in the on-site dormitory while 38 of them lived outside the facility in their own homes. As there was free social interaction among staff, all staff were regarded as potential contacts and were placed on 14 days quarantine from the start of the outbreak. No visitors were found to be in close contact with the index case in the last 14 days before the onset of symptoms.

### Epidemiological and Phylogenetic Characteristics of SARS-CoV-2 Transmission

The primary case of the nursing home is likely to be one of the infected HCW, Nurse S1, who had onset of symptom (headache) on March 17 while at work and had fever (38.2°C) on March 18 (**Figure 4**). Nurse S1 was on sick leave between March 18 and 24. In addition, the nurse did not have any recent travel history nor any known exposure to positive cases in the last 14 days before the first symptom onset. Notably, the husband (H1) of the nurse had traveled to Sri Lanka on February 26 and had arrived back in Singapore on March 14. As the 14-day stay home quarantine measure was only implemented for all inbound travelers as of 21 March 2020, the husband was not implicated with the home quarantine measure. However, a health advisory was provided to monitor their health and to seek medical attention if they display any respiratory symptoms. Therefore, the husband (H1) recalled that his first symptom onset was only on March 30. Both Nurse S1 and husband (H1) were diagnosed with COVID-19 on April 1 (Figure 2). All other nine household contacts of the two infected HCW were quarantined and were not COVID-19 positive.

**Figure 3:**
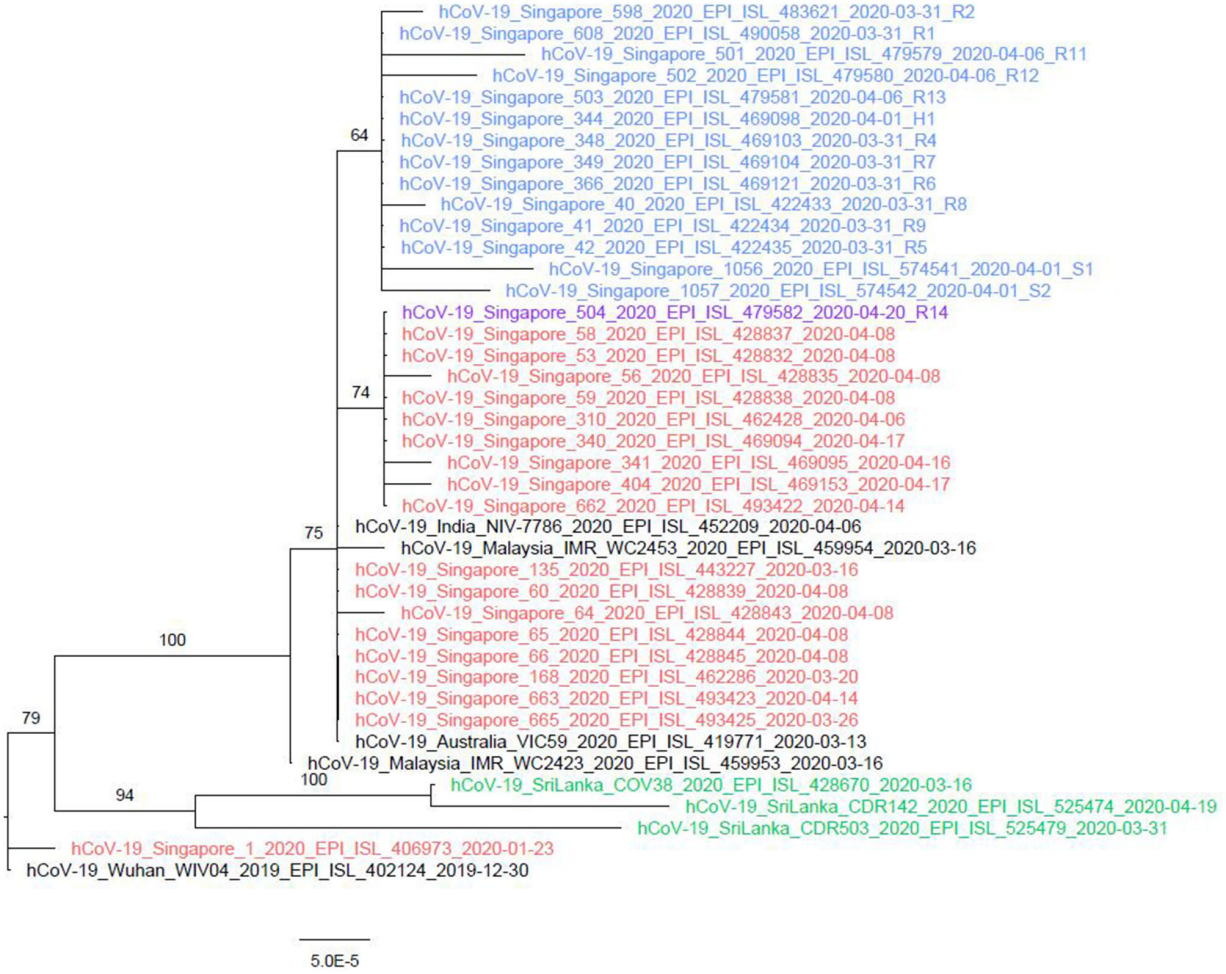
Phylogenetic tree of nursing home and the associated household member. The whole genome sequences of the cases from the nursing home (highlighted in blue) were closely clustered together, except for R14 (highlighted in orange). H1, the whole genome sequence of the husband of nursing staff S1 was genetically similar to the cases of the nursing home cluster and the India sequence. Viral quantity and quality were not high enough to conduct the whole genome sequencing for R3, R10, S1, and S2 successfully.

With the exception of R14, phylogenetic analysis of SARS-CoV-2 genome sequences obtained from all cases, including H1, was grouped into a single cluster (Figure 3, labeled in blue). This cluster was supported by a single mutation (T27588A) not found in other sequences in the database before the nursing home outbreak. In contrast, R14 bore a C23185T mutation which was absent as compared with other cases but was common among B.6 lineage sequences circulating in Singapore. This suggests that there might be more than one introduction of different strains of SARS-CoV-2 in this nursing home outbreak. However, definitive evidence was not available from this investigation, as only the symptomatic HCWs were screened and the full genomic sequences of R3, who was residing beside R14 in the same ward (Figure 1), was not successfully sequenced for comparison.

The nursing home outbreak sequences belonged to pangolin B.6 lineage (part of other GISAID clades not common globally), which predominantly circulated in Asia (primarily in India with 41% of all submitted strains between March and April 2020 classified as B.6, Malaysia 74% and Singapore 61% compared with only 1% globally). The extent of B.6 viruses circulating in Sri Lanka is unknown due to limited Sri Lanka sequences submitted into GISAID (14 as of September 7, 2020), even though the outbreak in Sri Lanka was identified on March 9, 2020. The phylogenetically closest strains from Sri Lanka in the database are classified as lineage B.4 and share a common ancestor with lineage B.6 characterized by variant G11083T. It is possible but not genetically proven that B.6 lineage strains circulated in Sri Lanka during the relevant time period.

**Figure 4:**
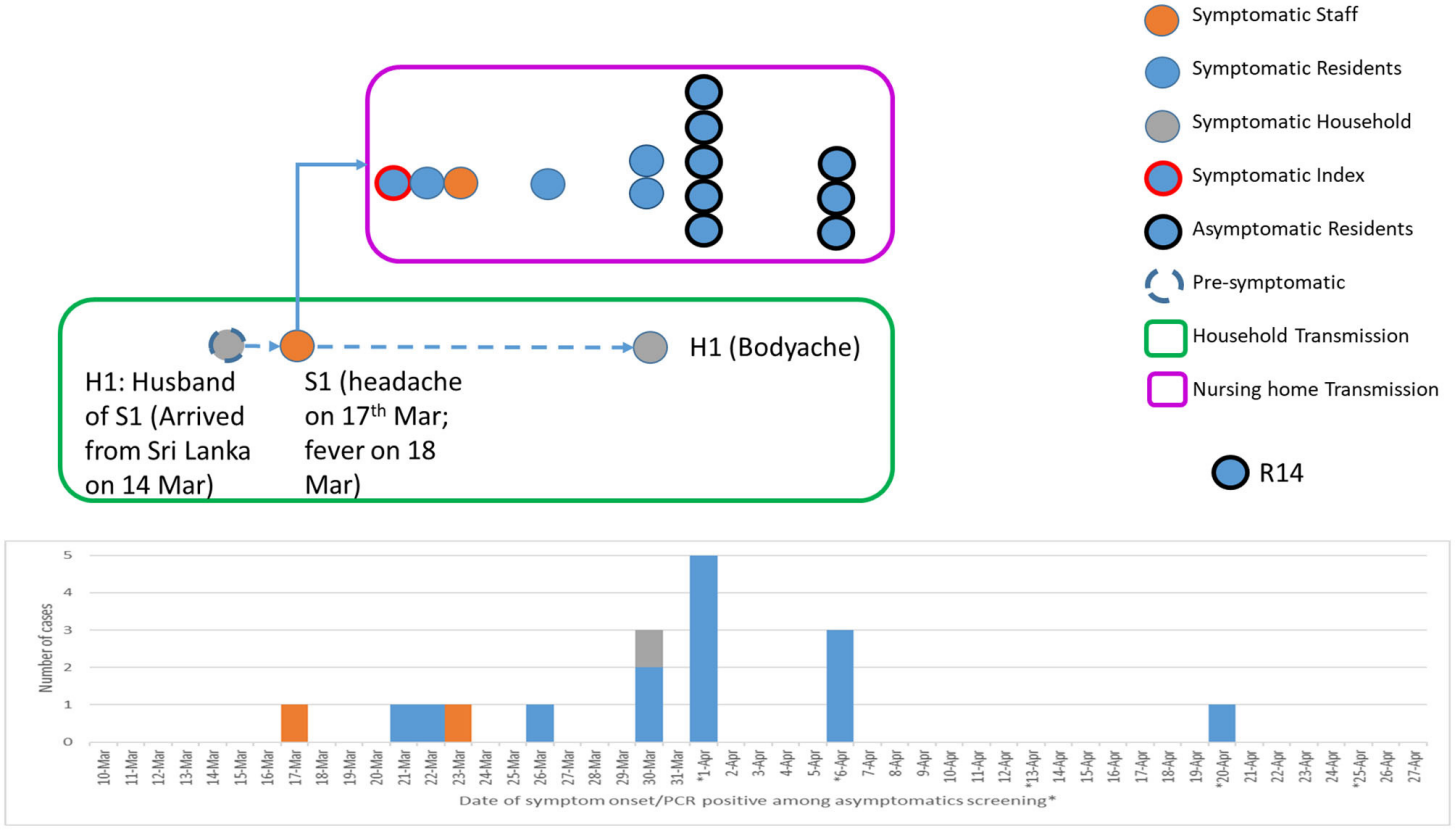
Epidemiological transmission and linkages between a household cluster and nursing home cluster. Pre-symptomatic household transmission occurred from H1 (husband of S1; travel history to Sri Lanka and returned on March 14) to S1 (wife of H1; staff nurse of a nursing home). This led to the introduction of the severe acute respiratory syndrome coronavirus 2 (SARS-CoV-2) transmission within the nursing home.

As such, there are two possibilities of transmission in the household. First, Nurse S1 had acquired the infection from her husband H1 pre-symptomatically, albeit less likely as the symptom onset was 13 days earlier than the husband and in view of the lack of other epidemiological exposure or contact with other confirmed cases (Figure 4). Second, Nurse S1 might have transmitted the virus to the husband (H1) after the onset of symptoms on April 17, 2020, with at least six epidemiologically linked secondary cases (Figures 3, 4).

## Discussion

The first COVID-19 outbreak in a nursing home in Singapore had an attack rate of 13% (14/108) and a CFR of 28.6% (4/14) among its residents. The CFR is significantly higher than the national rate of 0.1%, as of August 2020, and this is similar to nursing home outbreaks in Canada (19) and the United States (4). Residents residing in the nursing home tend to be elderly, frail, have multiple comorbidities, and impairment in function and cognition, which would have limited their ability to report symptoms accurately or they tend to have atypical presentation (20). The transmission rate may also increase if cognitively impaired residents continue to wander around the facility. By pre-emptively transferring these residents out of the facility for isolation, the further transmission was effectively curtailed.

Studies of other clusters have established pre-symptomatic and asymptomatic transmission of SARS-CoV-2 (2, 21, 22) and this is biologically plausible (23). Such transmissions pose challenges to the early detection and containment of outbreaks. Serial PPS, if resources permit, may allow for early detection of asymptomatic and pre-symptomatic patients to suppress transmission (2, 3, 24–26). Undetected infections have been shown to cause larger outbreaks in some clusters and mass screening rather than symptom-based testing is now widely accepted as the preferred strategy for the management of nursing home outbreaks (26–28). Strict compliance to IPC is critical as it may minimize the risk of pre-symptomatic transmission from HCWs to residents.

This outbreak only had two infected male residents compared with 12 female residents. The relative protection among the male wards was likely attributable to two infected HCWs who were managing the female wards, and the strategy of team segregation and cohorting which has been shown to be effective in limiting spread (29). However, this staff segregation needs to be maintained not only at work but also during mealtimes and social activities, and among the residential living arrangement of staff. Otherwise, inherent risks for cross infection will be present. Reducing the risk of infection among staff members has been associated with decreasing the risk of mortality among the residents (19).

Based on the epidemiological analysis, the HCW is potentially the source of transmission in this nursing home outbreak, despite adhering to PPE guidelines and taking sick leave. HCW who mingle in the community and engage in social interactions outside of work are a potential weak link in the prevention of outbreaks in a nursing home, especially when community spread is prevalent in the population. From May 2 to June 1, 2020, when community transmissions were evident in Singapore, the MoH implemented additional supportive measures to lodge most HCWs who were providing direct care to nursing home residents in hotels or dormitories on-site to minimize their exposure to infections in the community. HCWs underwent stringent health status checks and were required to declare family travel history as part of active surveillance. In addition, with effect from May 8, all healthcare staff (HCS) and residents at the nursing homes who developed acute respiratory symptoms were required to undergo testing for COVID-19. Strict implementation of infection prevention and control practices (30), improving air flow (31), active case finding through contact tracing with early detection, and isolation of close contacts and positive cases (32) as well as staff and associated households segregation plans can significantly reduce the morbidity and mortality of residents in a nursing home outbreak. On another perspective, household-based nursing care may not be the best alternative approach to reduce the risk of spread among these vulnerable older adults, especially when residing with working-age adults who have a high risk of exposure from their social, workplace, and community contacts (33). Furthermore, a safe and effective vaccine against SARS-CoV-2 should be prioritized among the HCS and residents in nursing homes whenever earliest possible.

There are some limitations to this investigation. The activities in the last 14 days and onset of symptoms of the detected cases were based on recall and hence, there was potential recall bias. However, the shift work of nursing staff and activities of residents as well as travel history were well-documented. As only symptomatic HCWs were screened, the possibility of more than one introduction of SARS-CoV-2 cannot be completely excluded. However, the likelihood is low as infection prevention and control measures were further strengthened after the index case was identified. Point prevalence survey was only done on all residents and only among symptomatic HCW. Moreover, seroprevalence was not performed, due to the lack of a validated serology platform, to assess the overall COVID-19 positivity rate of the nursing home during the early phase of a pandemic. Hence, there is a potential underestimation of attack rate and overestimation of CFR. Although the availability of whole genomic sequencing provides additional evidence to strengthen the epidemiological linkages, the incomplete whole genome sequences of the two HCW cases and the limited submission of genetic sequences from Sri Lanka potentially underestimate the likelihood of the phylogenetic linkages between Sri Lanka and the nursing home outbreak.

## Conclusion

The nursing home is vulnerable to SARS-CoV-2 introduction and transmission among the infected nursing staff who have direct household and community contacts. There is potentially high case fatality and secondary attack rates in nursing homes without a regular serial screening of SARS-CoV-2 among residents and HCS.

## Data Availability Statement

The datasets presented in this study can be found in online repositories. The names of the repository/repositories and accession number(s) can be found in the article/Supplementary Material.

## Ethics Statement

This study was conducted in response to a national public health emergency. All above-mentioned activities and measures were performed in accordance with the guidelines and guidance of the laboratory safety, infection prevention and control, and clinical management circulars approved by the Ministry of Health (MoH) for the management of the COVID-19 outbreak as well as the Infectious Diseases Act enacted by Parliament in Singapore to safeguard the public health in Singapore. Informed consent was obtained from all subjects under the Infectious Diseases Act.

## Author Contributions

HT: conceptualization. JP, HT, TM, SO, MC, IL, VK, PO, SV, LC, HR, JC, and VL: data curation. JP, HT, TM, SO, SM-S, FL, MC, IL, SV, DF, LC, JC, and VL: formal analysis. JP, HT, MC, IL, VK, PO, SV, DF, LC, HR, JC, and VL: investigation. JP, HT, TM, SO, SM-S, FL, PO, SV, DF, LC, HR, JC, and VL: methodology. JP, HT, MC, IL, VK, PO, HR, JC, and VL: project administration. JP, TM, SO, SM-S, FL, HR, JC, and VL: resources. JP, TM, SO, SM-S, and FL: software. SM-S, MC, IL, LC, HR, JC, and VL: supervision. JP, HT, TM, SO, SM-S, FL, MC, IL, PO, SV, DF, LC, JC, and VL: validation. JP, HT, TM, SO, SM-S, and FL: visualization. JP and HT: writing–original draft. JP, HT, TM, SO, SM-S, FL, MC, IL, VK, PO, SV, DF, LC, HR, JC, and VL: writing–review and editing. All authors contributed to the article and approved the submitted version.

## Funding

This study was supported by the Ministry of Health, Singapore and Saw Swee Hock School of Public Health, National University of Singapore as part of the national response against the COVID-19 outbreak in Singapore.

## Author Disclaimer

Any opinions, findings, and conclusions, or recommendations expressed in this report are those of the authors and do not reflect the views of the MoH, AIC, NCID, NUS, and TTSH in Singapore.

## Conflict of Interest

The authors declare that the research was conducted in the absence of any commercial or financial relationships that could be construed as a potential conflict of interest.

## Publisher's Note

All claims expressed in this article are solely those of the authors and do not necessarily represent those of their affiliated organizations, or those of the publisher, the editors and the reviewers. Any product that may be evaluated in this article, or claim that may be made by its manufacturer, is not guaranteed or endorsed by the publisher.
